# A secure SNP panel scheme using homomorphically encrypted K-mers without SNP calling on the user side

**DOI:** 10.1186/s12864-019-5473-z

**Published:** 2019-04-04

**Authors:** Sungjoon Park, Minsu Kim, Seokjun Seo, Seungwan Hong, Kyoohyung Han, Keewoo Lee, Jung Hee Cheon, Sun Kim

**Affiliations:** 1 Department of Computer Science and Engineering, Seoul National University, Seoul, Republic of Korea; 2 Interdisciplinary Program in Bioinformatics, Seoul National University, Seoul, Republic of Korea; 3 Hyperconnect Inc, Seoul, Republic of Korea; 4 Department of Mathematical Sciences, Seoul National University, Seoul, Republic of Korea; 5 Bioinformatics Institute, Seoul National University, Seoul, Republic of Korea

**Keywords:** SNP panel, Homomorphic encryption, K-mer, Genomic security, Genomic privacy

## Abstract

**Background:**

Single Nucleotide Polymorphism (SNP) in the genome has become crucial information for clinical use. For example, the targeted cancer therapy is primarily based on the information which clinically important SNPs are detectable from the tumor. Many hospitals have developed their own panels that include clinically important SNPs. The genome information exchange between the patient and the hospital has become more popular. However, the genome sequence information is innate and irreversible and thus its leakage has serious consequences. Therefore, protecting one’s genome information is critical. On the other side, hospitals may need to protect their own panels. There is no known secure SNP panel scheme to protect both.

**Results:**

In this paper, we propose a secure SNP panel scheme using homomorphically encrypted K-mers without requiring SNP calling on the user side and without revealing the panel information to the user. Use of the powerful homomorphic encryption technique is desirable, but there is no known algorithm to efficiently align two homomorphically encrypted sequences. Thus, we designed and implemented a novel secure SNP panel scheme utilizing the computationally feasible equality test on two homomorphically encrypted K-mers. To make the scheme work correctly, in addition to SNPs in the panel, sequence variations at the population level should be addressed. We designed a concept of Point Deviation Tolerance (PDT) level to address the false positives and false negatives. Using the TCGA BRCA dataset, we demonstrated that our scheme works at the level of over a hundred thousand somatic mutations. In addition, we provide a computational guideline for the panel design, including the size of K-mer and the number of SNPs.

**Conclusions:**

The proposed method is the first of its kind to protect both the user’s sequence and the hospital’s panel information using the powerful homomorphic encryption scheme. We demonstrated that the scheme works with a simulated dataset and the TCGA BRCA dataset. In this study, we have shown only the feasibility of the proposed scheme and much more efforts should be done to make the scheme usable for clinical use.

## Background

Single Nucleotide Polymorphism (SNP) is crucial information in medical sciences than ever before. A single aberrant nucleotide variation can incur dysfunction in a biological process, affecting individual vulnerability to certain diseases. Hence, SNP existence can be utilized to diagnose diseases. Sometimes, SNPs help determine effective treatment, especially in cancer. From The Cancer Genome Atlas (TCGA) project, numerous driver mutations are reported in many cancer types [1] and panels using the curated mutation information have been developed [2]. Furthermore, genetic disorders in Mendelian diseases are very well studied [3] and thus SNP detection can be directly translated into the contribution to actual medical applications. Because of the importance of SNPs to disease, the US NIH has compiled a database called ClinVar [4].

The utility of SNPs goes beyond the medicine domain. People are diverse in the genomic content, thus the difference in genomic content among people can be used to identify a specific person. For such reason, SNPs are often used for legal and forensic purposes. There are Direct-to-Customer SNP kits that are designed for non-medical use such as pedigree search. More people measure their genomes and use the information for various purposes.

As genomic data are widely used and disclosed, a potential threat to genomic privacy is a serious problem. DNA sequence is a blueprint of a human being since the information includes not only medical conditions but to the extent of even reconstructing facial model which is one of the most sensitive information of human being [5]. Our knowledge on the human genome is very limited as of now and much more information encoded in the genome will be disclosed over the years. Thus, genomic information is critical and will cause severe damage to individuals if leaked. This could also lead to a social crisis because genomic data can be used to justify discrimination among people. Unlike other confidential data, genomic data are innate and immutable, making the damage irreversible and permanent throughout one’s lifetime. Due to its far-reaching and sensitive nature, the genomic data is prone to be monetized. For these reasons, It is very important to protect the genomic information from hackers, insurance establishments, hospitals, pharmaceuticals, government and all the possible threats yet to come.

Among recent measures to protect data, homomorphic encryption is a technology that gains attention recently. It refers to an encryption scheme that allows the third party to perform computations while not knowing any content of the original source data or the private key. If such computation includes addition and multiplication, then theoretically all computations can be performed and that is called fully homomorphic encryption. The result of the computation is also returned encrypted, thus the third party can provide its inference based on the data and at the same time cannot know the data content or perform inference at all. The first fully homomorphic encryption scheme is proposed by Gentry at 2009 [6], and continually improved its efficiency.

For example, consider that Alice is running a company and Bob provides a cloud storage service. Alice stores her encrypted data on Bob’s cloud. When Alice wants to compute aggregate data, an average revenue per month for instance, she has to decrypt the data if it is traditionally encrypted. Decrypting on the cloud storage may reveal both the private key and the source data to Bob. Downloading the encrypted data to local and decrypting it goes against the purpose of utilizing cloud service in the first place. This is when homomorphic encryption comes useful. If the data is homomorphically encrypted, Alice does not have to decrypt the source data on Bob’s cloud to do the computation needed. Instead, the encrypted form of aggregate data can be computed as ciphertexts and downloaded to Alice’s computer, and then safely decrypted. This way, Alice can exploit Bob’s resource to compute the average revenue without giving any information to Bob.

In more formal notation, an encryption scheme is additive homomorphic encryption if and only if

$$\forall p ~ \forall q ~ \exists \odot : \mathcal{E} (p) \odot \mathcal{E} (q) = \mathcal{E} (p + q) $$

given plaintexts *p*, *q*, and $ \mathcal {E} (\cdot) $ an encryption procedure. $ \mathcal {E} (p + q) $ denotes a ciphertext that can be decrypted to plaintext *p*+*q* with the private key. Multiplicative homomorphic encryption works likewise.

### Related work

Given the importance of genome information, there has recently been active research on genome security. Naveed et al. [7] presented the history of genomics and related privacy issues including the homomorphic encryption. Dowlin et al. [8] also demonstrated how homomorphic encryption and security could be used in the fields of bioinformatics. In detail, we categorize recent genomic security research works into three major groups: (1) differential privacy, (2) secure system design with traditional encryption scheme and (3) homomorphic encryption scheme.

Differential privacy includes de-identification, which refers to making genomic data unidentifiable by either anonymizing or discarding personally differential information. This aims to perturb the information so that any leak of data itself would not possibly lead to identifying the patient. However, it is shown in many papers that generic de-identification techniques are not powerful enough to prevent reconstructing identity [9–13]. To mitigate the risk, many improvements were made based on the domain knowledge of the genomic data [14–19].

The secure system design with traditional encryption focuses on controlling the flow of sensitive information. It relies on secret sharing of private keys with multiple parties that do not collude to ensure the confidentiality of the data. Canim et al. [20] suggested secure operations based on a cryptographic coprocessor. Kamm et al. [21] adopted multiple third parties to securely perform GWAS analyses in a distributed way. On the other hand, Xie et al. [22] proposed a statistical approach called meta-analysis to recall aggregate features with reduced privacy risks. In the work of Wang et al. [23], secure and efficient computation of genomic edit distance and querying similar sequence based on that is introduced.

The application of homomorphic encryption dates back only to few years since it is a new technology. Troncoso-Pastoriza et al. [24] proposed error resilient private string search algorithm that is specially designed for DNAs using homomorphic encryption. Kantarcioglu et al. [25] also adopted homomorphic encryption to securely share the aggregate data of genome sequence among researchers. Ayday et al. [26, 27] proposed methods to query the disease susceptibility with clinical data encrypted. More recently, Kim et al. [28] showed that homomorphic encryption can be used to obtain minor allele frequencies, *χ*^2^ statistic in GWAS and edit distance of sequences in a secure way. Lu et al. [29] and Zhang et al. [30] encrypted phenotype and genotype homomorphically and then was able to infer typical GWAS statistic. On the other hand, Wang et al. adopted homomorphic encryption on rare variants to perform homomorphic exact logistic regression [31]. Raisaro et al. [32] showed retrieving aggregate data computed under homomorphically encrypted data that is deployed to real world application on i2b2 data warehouse. Jagadeesh et al. [33] also have shown secure SNP data sharing between hospitals to induce meaningful inference of disease. Other most recent works on homomorphic encryption include the works of Jacquez et al. [34], Ghasemi et al. [35], and Cheng *et al* [36].

As described above, research on genomic privacy has been active and advanced significantly over the years. However, current research has been conducted assuming situations with some compromises. De-identification more or less manipulates the content of data and thus has possibility to contaminate the original information. The secure system design distributes the secret key and the computation to multiple third parties. However, either the trust or the resources for computing may not be available in reality. If the third parties are untrustworthy, they may collude to jeopardize the private system. Likewise, the shortage of resources such as storage, bandwidth and processing power is critical for such system to maintain.

Even the works with homomorphic encryptions have their own limitations, due to its inefficient nature. Most applications [25*–*27*,*^30^*–*32] encrypt a clean, annotated SNP existence information on the user side or clinic data as a plaintext. In this case, SNP calling should be done on the user side and then the SNP calling information is sent to the hospital. Some applications send sequences to the hospital [25*–*29*,*^37^]. In this case, the querying result is limited to aggregate genomic data and clinical data. How to use these techniques for a secure SNP panel has not been explored.

### Motivation and contribution

**Fig. 1:**
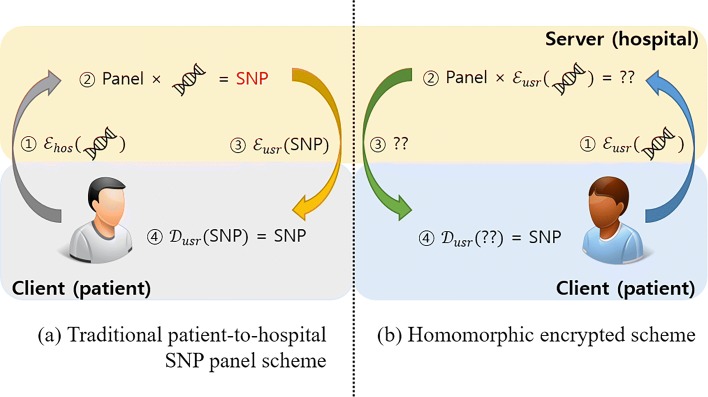
The traditional and homomorphic SNP detection scheme. In this figure, $\mathcal {E}(\cdot)$ and $\mathcal {D}(\cdot)$ denote encryption and decryption respectively. Encryption needs public key while decryption needs private key. The subscript refers to the owner of the private key. For example, $\mathcal {E}_{hos} (\cdot)$ means the data is encrypted with hospital’s public key thus only the hospital can decrypt it. **a** demonstrates traditional way of detecting SNP. ① The patient gives the hospital the raw sequence. In order to protect data from being stolen in the middle, the sequence is encrypted using the hospital’s public key. ② The hospital decrypts patient sequence and performs computation to detect SNP. ③ The hospital returns the SNP existence information encrypted with the patient’s public key to the patient. ④ The patient can decrypt and get the SNP information. **b** on the other hand, demonstrates the same SNP detection scheme in homomorphic way. ① The patient sends one’s encrypted raw sequence but this time with the public key. ② Due to its homomorphic property, the hospital can perform computations on the sequence only with the public key, without decrypting the sequence. ③ The result is acquired in encrypted form, and the hospital returns the result to patient not knowing its content. ④ The patient can decrypt the result in secure environment

In this paper, we propose a patient-to-hospital SNP panel scheme and its architecture is illustrated in Fig. 1. In our two party model, **the patient** has one’s raw sequence and the private key, and **the hospital** has computing power and the SNP panel. SNP panel refers to a tool owned by the hospital that can find the specific combination of SNPs.

A possible scenario is that the patient sends the raw sequence to the hospital to detect SNP existence. In order to protect data from being stolen in the middle, the transferring raw sequence must be encrypted. In traditional patient-to-hospital model, the sequence is encrypted using the hospital’s public key. This method reveals the patient’s raw sequence. On the other hand, with homomorphic encryption, the patient can send the raw sequence encrypted with his/her own public key. Plus, the homomorphic nature of encryption allows the hospital to match encrypted sequence with its SNP panels without the private key or knowing the raw sequence.

Another thing to note is that, homomorphic encryption is slow and expensive that it cannot be applied to raw sequence for practical usage due to its computational inefficiency. In addition, although additive and multiplicative homomorphic encryption schemes is considered fully homomorphic in theory, its functionality is limited in reality due to their computationally demanding nature. Thus, using domain knowledge of genomic sequence, we devise a patient-to-hospital communication protocol onto which homomorphic encryption is applicable in order to securely detect SNPs without revealing the patient’s raw sequence and the hospital’s SNP panel assets to each other.

Thus, we propose a secure SNP panel scheme using homomorphically encrypted K-mers without SNP calling on the user side. The major challenge is that there is no known algorithm to align two homomorphically encrypted sequences as whole. The basic idea to overcome this challenge is to utilize the equality testing operation on two homomorphically encrypted K-mers that is computationally feasible under the homomorphic encryption scheme. The major features of our scheme is as below:

1. We introduce a secure scheme that uses homomorphically encrypted K-mer, a short subsequence of the raw sequence, and show that encrypted K-mers can detect SNPs as good as the raw sequence does under certain conditions. With properly tuned value of K, exploiting K-mer can achieve small error bounds and practical runtime at the same time.
2. Our method considers genome variations among individuals. We assayed TCGA breast cancer patient data to estimate individual variation ratio. We further define and compute false positive rate and false negative rate of our SNP detection scheme and either suggest a method to control or show that the error is bounded to an ignorable value.
3. Our contribution is also to the extent of providing a SNP panel design guideline. When a hospital selects SNP residues for the diagnosis of a certain disease and wants to use our secure SNP panel scheme, our method suggests guidelines on which SNP residues under consideration can be used.

## Methods

### Data description and panel preprocessing

In actual clinical case, a SNP *panel* consists of multiple SNP *residues*, where each SNP residue corresponds to a pre-determined disease-associated SNP residue (Fig. 1). A targeted DNA sequencing is then performed to assess SNP status of each residue.

For our study, we generated a synthesized dataset that simulates the aforementioned case. In the dataset, the SNP residues are randomly sampled from coding sequences of the hg19 reference genome [38], where refSeq gene annotation is used to specify coding regions (downloaded from UCSC genome database [39]). First, we chose 1000 SNP residues sampled from the hg19 genome, then randomly combined them into various size of panels. As a result, we generated panels having 10, 20,..., 100 residues randomly selected from the pool of 1000 SNP residues. Hence, 10 different SNP panels of size ranging from 10 to 100 were generated.

After combining 10 different SNP panels, we simulated a massive parallel sequencing data (or DNA-seq) using WgSim. From the 1300 bp length flanking sequences (650 bp for each side) from each SNP residue, short-read sequences (151 bp × 2) were simulated. Here, the 1300 bp and “151 bp × 2” parameters were set to simulate the actual targeted short-read sequencing condition. The exact parameter for WgSim is “-e 0 -1 151 -2 151 -r 0 -R 0 -X 0 -S 0 -N [VAR]”, where all the parameters for random sequencing errors are set to none and the throughput parameter “-N” depends on the size of each panel. The sequencing depth was set to 10, meaning that 10 sequencing reads are expected to cover each SNP residue in average. So, to cover more residues, it requires more sequencing reads to simulate (hence larger “-N”). Lastly, each residue has 50% chance of nucleotide substitution to simulate SNPs by design. For instance, if we check any SNP residue, we can expect to find 5 reads having reference allele and other 5 reads having variant allele as planted. In summary, we generated 10 DNA-seq data corresponding to 10 SNP panels having different combination of SNP residues, each having average 50% substitution rate.

**Fig. 2:**
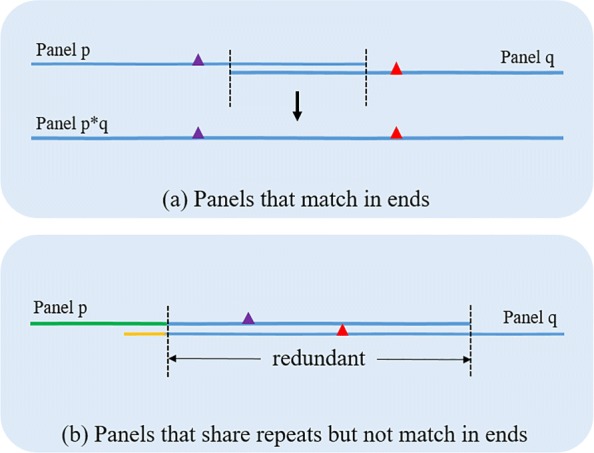
Two possible cases where different SNP residues have an overlap in their flanking sequences. There can be two different cases where an overlapping occurs between flanking sequences of two different SNP residues. **a** Fully overlapped without mismatch, **b** Partially overlapped with mismatch. In case of (**a**), two SNP residues are merged into a single sequence to prevent unnecessary long search for K’s. However, in case of (**b**), we randomly chose one residue and discarded the other one

There could be unwanted overlap of flanking sequences between two different SNP residues. The overlap between two SNP residues would require a large value K. Therefore, we either merge the overlapped residues or discard one of them. This problem is described in Fig. 2. If two SNP residues are fully overlapped without mismatch, we merge them into a single entity having extended flanking sequences to prevent unnecessary long search for Ks. And if not, we randomly chose one residue and discarded the other one. To avoid promiscuous merging, we set the cutoff for fully overlap situation to 100 bp. Assuming a uniform random distribution, the probability that two flanking sequences overlap 100 bp by chance is 1−(1−2·4^−100^)^2^. The computed p-value is negligible. Therefore, it is safe to merge the residues satisfying this condition. However, if an extreme case occurs that flanking sequences of two SNP residues overlap more than 150 bp, which is longer than short-read length, we treat them same as the case of partial overlap situation, hence they are discarded.

The handling of overlapping sequence enables that, even if more than two SNP residues exist near, possibly within the range of K so that there exists a K-mer which has both SNPs, the proposed SNP panel scheme finds both SNPs correctly. It only has to enumerate all possible K-mers and perform equality test on the user K-mers with corresponding SNP labels.

Now we have a panel that will be used for testing our secure SNP scheme. The panel should be designed by doctors for a specific disease. What we have shown is a computational scheme to test whether a panel with many residues can be used with our secure SNP panel scheme that is proposed in this paper.

### Workflow: a bird’s eye view

The goal of this paper is to propose a patient-to-hospital secure communication protocol such that a patient would conceal its genome sequence while requesting SNP detections to a hospital with a panel. Specifically, we are interested in separating access between to the SNP panel at the hospital and to the genomic sequence of the patient. The SNP panel information is an asset of the hospital which may not be revealed to the public. On the other hand, the user does not want the hospital to know one’s sequence. The goal is to protect both.

**Fig. 3:**
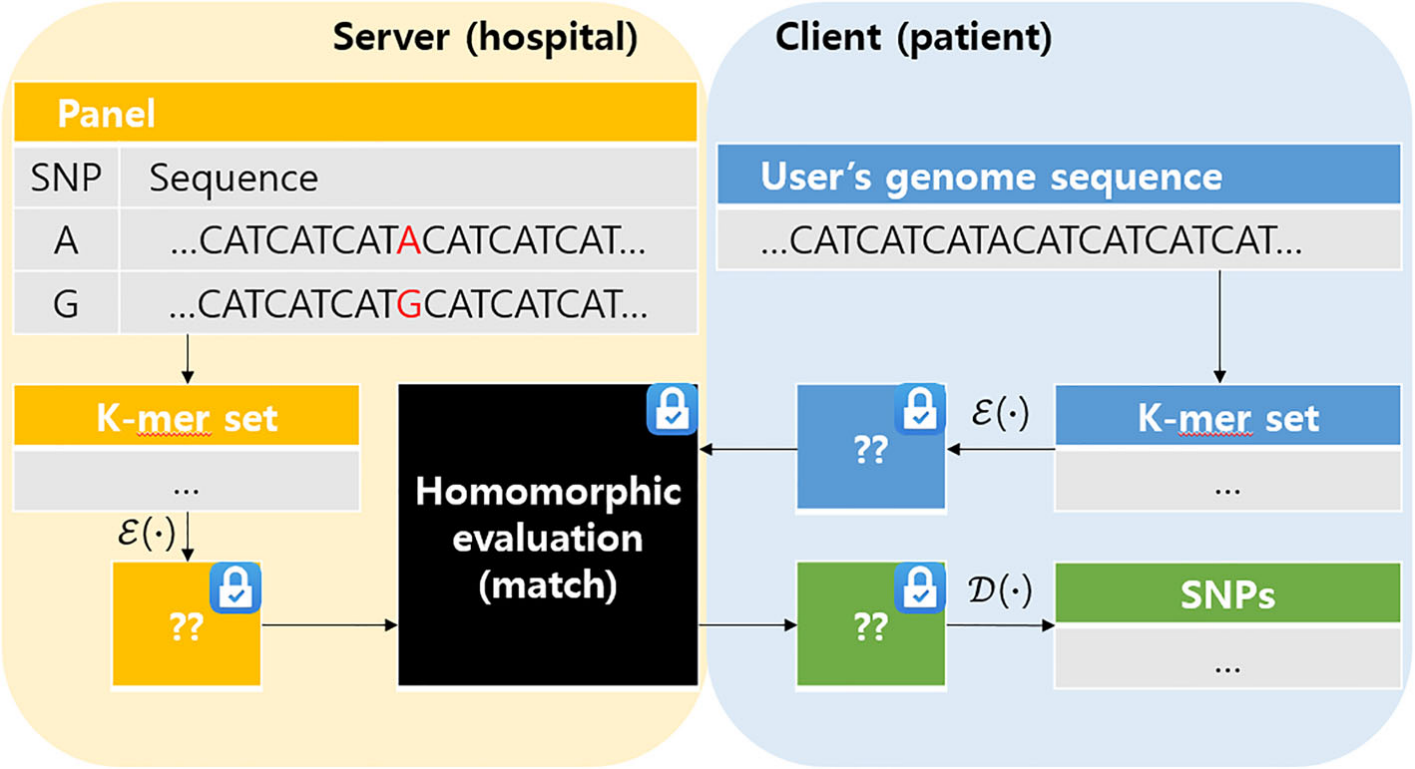
The overall architecture of our new SNP detection scheme. This figure depicts the general process of proposed SNP detection scheme. The set of panel sequences noted in yellow and the patient genome sequence noted in blue are chunked down into K-mers, respectively. After we encrypt K-mers, the client-side patient’s encrypted K-mers are transmitted to the server. The black box depicted in the middle denotes the homomorphic evaluation procedure. Here, we check the matching correspondence between any member in panel K-mer set and in the patient K-mer set. The result noted green is returned to the patient. The patient can decrypt the result with the private key securely on the client-side environment

In our scheme illustrated in Fig. 3, the patient has one’s raw genomic sequence and the hospital has panel sequence. To apply homomorphic encryption to sequences, we chunk both the raw sequence of the patient and the panel sequence of the hospital into K-mers. In other words, we get K-mers from substring of the sequences with a sliding window of stride 1, size K. What we want to do is encrypt K-mers and perform **homomorphic equality tests** on two K-mers. The patient would receive an encrypted result of SNP calling from the hospital. If a K-mer from patient sequence matches a K-mer from the panel sequence annotated with a specific SNP, the patient can tell which SNP one has. Further medical diagnosis can be done based on the test result.

Note that the patient encrypts the K-mers and holds the private key so that the hospital does not gain any information about the patient’s sequence. Meanwhile, the computation is done on the hospital’s side, securing SNP panel assets from leakage.

### K-mer design

Our SNP detection scheme works under specific conditions. To determine SNP existence by performing K-mer equality testing, the corresponding K-mer should be unique throughout the whole SNP panel sequences. Otherwise, K-mer matching cannot guarantee the existence of SNP. Thus the core idea of K-mer design is to set K long enough for all K-mers that contain any panel SNP to be distinguishable. However, while long K ensures unique K-mers, longer K-mers will be computationally expensive.

Therefore, given a panel we computed panel-specific minimum value of K such that all K-mers in the panel that have a SNP are distinct. To achieve this goal, first K-merize all panel sequences and group them into two sets: (A) K-mers with SNPs and (B) K-mers without. Then, following conditions must hold for K-mers with SNPs to be distinct.

1. K-mers in set (A) are unique (have no duplicate)
2. K-mers in set (B) do not appear in set (A)

Among the Ks that satisfy the above conditions, the minimum value is chosen.

### False positive/negative errors

In our system, K of K-mer is defined to make all K-mers distinguishable and thus no false positive or negative errors. However, we assumed that individual patient sequence derived from SNP panel varies only at the interested SNP residue and the flanking sequences are identical for all patients. This is not the case when *the variations at the population level* are considered. An actual sequence derived by SNP panel may present unannotated deviations from what is known to the panel, other than SNP residues in it. Examples include point mutation, individual genome variation and sequencing errors. Presence of such deviations may result in false positive and false negative errors. False positive error occurs when the scheme identifies a K-mer and declares a SNP existence but actually the patient does not have one. False negative error occurs when the patient indeed has a SNP yet the scheme fails to detect one. We henceforth refer to point deviation as a single nucleotide deviation from intended panel sequence, resulting from either point mutation, individual variation or sequencing error.

False positive occurs when the determined K-mer did not originate from the flanking sequence around the found SNP. Rather, it originated from other irrelevant parts of patient genome sequence that correspond to any other SNP residue in the processing panel. To deal with false positive errors, we devised **Point Deviation Tolerance (PDT) level**. Previously in the process of computing K, we had two conditions on K-mers. Both conditions utilized the equality test to check uniqueness of K-mers in (A) and exclusiveness of K-mers in (A) over those in (B). We generalize the equality test to the hamming distance check with its lower bound being PDT. In other words, we apply strict rules and regard similar sequences ambiguous. Here, being similar is defined by PDT point deviations. The uniqueness condition is the specific case of PDT being 0. Thus we can rewrite the K-mer conditions as below:

1. K-mers in set (A) are distinct (any pairs’ hamming distance greater than PDT)
2. K-mers in set (B) are distinct from K-mers in set (A)

Given PDT, one can determine a minimum value K satisfying the updated conditions. PDT works as a safety margin to K-mer ambiguity. The point deviation, namely the sum of point mutations, individual variation and sequencing errors, is allowed up to maximum PDT. Therefore, if the aggregate point deviation occurs less than or equal to PDT, false positive cases do not appear.

On the contrary, we cannot prevent false negative cases. False negative happens when the system cannot determine a SNP when it is truly in the patient’s sequence. The major cause of this is also the point deviation in the sequences flanking a SNP. It is infeasible to prepare all the variant K-mers as we did to cope with the false positive errors. In this paper, we assayed TCGA BRCA data to determine the distance distribution of somatic mutations among patients. Based on the data, we estimate the probability of a point mutation lying on K-mers to estimate false negative errors.

### RLWE cryptosystem

In this paper, we used HEAAN (Homomorphic Encryption for Arithmetic of Approximate Numbers) library to implement the equality test on two homomorphically encrypted K-mers. HEAAN is based on RLWE (Ring-Learning With Error) encryption scheme. RLWE is a variation of LWE (Learning With Error) problem, which is a lattice-based cryptography. LWE exploits its hardness assumption to ensure security which follows:

$$ a\! \leftarrow\! {\mathbb{Z}}^{n}_{q}, s \!\leftarrow\! {\mathbb{Z}}^{n}_{q}, e \!\leftarrow\! {\chi}^{n}, r_{1}, r_{2} \!\leftarrow\! {\mathbb{Z}}^{n}_{q} : (a, \langle a, s \rangle + e) {\approx}^{c} (r_{1}, r_{2}) $$

In other words, for some secret key *s* and some error distribution *χ*, the relation between *a* and 〈*a*,*s*〉+*e* are computationally indistinguishable from random numbers. RLWE uses polynomial integer rings instead of vectors. Namely, ${\mathbb {Z}}^{n}_{q}$ s are replaced with ${\mathbb {Z}}_{q}[\!X]/{\Phi }_{m}(X)$ for *n*=*ϕ*(*m*), where *Φ*(·) is cyclotomic polynomial and *ϕ*(·) is Euler’s phi function. RLWE is estimated to achieve equal or less level of security compared to LWE. Other parameters for the scheme are *p* for message modulus, *q* ciphertext modulus and ring $R = \mathbb {Z}/{\Phi }_{M}(X)$ for integer *M*. We further denote by *R*_*q*_=*R*/*qR* and *χ* for error distribution. The scheme for cryptography used throughout this paper is described in detail below. SKGen(params) Choose random *s*(*X*)←*χ*, and set ${sk} = \vec s = (1, s)\in R_{q}^{2}$. PKGen(params, sk) Choose random *a*(*X*),*a*^′^(*X*)←*R*_*q*_, *e*(*X*),*e*^′^(*X*)←*χ*, and set *b*(*X*)=−*a*(*X*)*s*(*X*)+*pe*(*X*)∈*R*_*q*_ and *b*^′^(*X*)=−*a*^′^(*X*)*s*^′^(*X*)+*pe*^′^(*X*)∈*R*_*q*_. The public key is *pk*=(*b*,*a*)∈*R*^2^ and the evaluation key is *evk*=(*b*^′^+*ps*^2^,*a*^′^)∈*R*_*q*_. Enc(pk, *m*∈*R*_*p*_) Choose *v*(*X*),*e*_0_(*X*),*e*_1_(*X*)←*χ* and let *c*_1_(*X*)=*m*(*X*)+*v*(*X*)*b*(*X*)+*pe*_0_(*X*),*c*_2_(*X*)=*v*(*X*)*a*(*X*)+*pe*_1_(*X*). Return $\vec c = (c_{1}, c_{2}) \in R_{q}^{2}$. Dec(sk, $\vec c$) Return $[\langle \vec c, {sk} \rangle $. Add($\vec c_{1}$, $\vec c_{2}$) Return $\vec c_{\text {add}} = \vec c_{1} + \vec c_{2}$. Mult($\vec c_{1}$, $\vec c_{2}$, *evk*) For $\vec c_{1} = (b_{1}, a_{1})$ and $\vec c_{2} = (b_{2}, a_{2})$. Return $\vec c_{\text {mult}} = (b_{1}b_{2}, b_{1}a_{2} + b_{2}a_{1}) + a_{1}a_{2}\cdot evk \in R_{q}$.

RLWE-based homomorphic encryption supports batching (or SIMD) encoding and data array movement. If we call the each element of data array as slot, the scheme has permutation of message slots. This functionality can be used to make our homomorphic evaluation algorithm more efficient and split and merge DNA information. KeySwitchingMatrixGen(params, *sk*_1_→*sk*_2_) KeySwitch(*c*, ${KS}_{{sk}_{1} \rightarrow {sk}_{2}}$) KeySwitchingMatrixGen (*params*,*sk*(*X*^*k*^) → *sk*(*X*))Automorphism$\left (\textit {c}, X \rightarrow X^{k},\ {KS}_{{sk}\left (X^{k}\right) \rightarrow {sk}(X)}\right)\phantom {\dot {i}\!}$

### Data encoding and encryption

To perform equality tests of K-mers in numerical system, we regard each K-mer as a quaternary number via mapping each nucleobase A, C, G, T to 0,1,2,3, respectively and encode K-mers into integers. In this view, for example, if "GACT" is a K-mer of length 4, then it corresponds to *K*=2013_(4)_=2×4^3^+0×4^2^+1×4^1^+3=135.

However, encoding DNA sequence of length *L* wholly as an integer is inefficient when *L* is large, requiring a huge set of scheme parameters. To achieve a better performance, we suggest a method of breaking K-mers into smaller blocks of same length and performing equality tests for each block simultaneously. Henceforth for *N* user-side K-mers and *M* panel-side K-mers divided into *B* blocks respectively, we denote the data as following:

- *n*-th user-side K-mer : $K_{usr}^{(n)}$, or *K*^(*n*)^ when obvious. (*n*=0,1,⋯,*N*−1)
- *m*-th panel-side K-mer : $K_{ref}^{(m)} (m = 0, 1, \cdots, M-1)$
- *b*-th block of $K_{usr}^{(n)}$ : $K_{usr}^{(n)}[\!b]$, or *K*^(*n*)^[ *b*] when obvious. (*b*=0,1,⋯,*B*−1)
- size of a block: L

Once the set of user-side K-mers is ready, we can encode all user K-mers into *B* vectors. Specifically, *b*-th blocks of *N* user-side K-mers {*K*^(*n*)^[ *b*]}_*n*∈[*N*]_ are encoded into a single vector $\vec {v}_{b} = \left (\vec {v}_{b}[\!0], \cdots, \vec {v}_{b}[\texttt {\!slots} - 1]\right)$, where each component of $\vec v_{b}$ is defined as

$$ \vec{v}_{b}[\!i]= \left\{ \begin{array}{ll} 1, & \text{if } i=K^{(n)}[\!b]\cdot N + n\text{ for some }n\in[\!N]\\ 0, & \text{otherwise } \end{array}\right.. $$

Here, we choose the dimension of vector slots by smallest power of 2 which does not exceed 4^*L*^·*N*, or the maximum of *K*^(*n*)^[ *b*]·*N*+*n*. It is noteworthy that *K*^(*n*)^[ *b*]·*N*+*n*=*K*^(*m*)^[ *b*]·*N*+*m* if and only if *n*=*m*, since *n*,*m*<*N*.

**Fig. 4:**
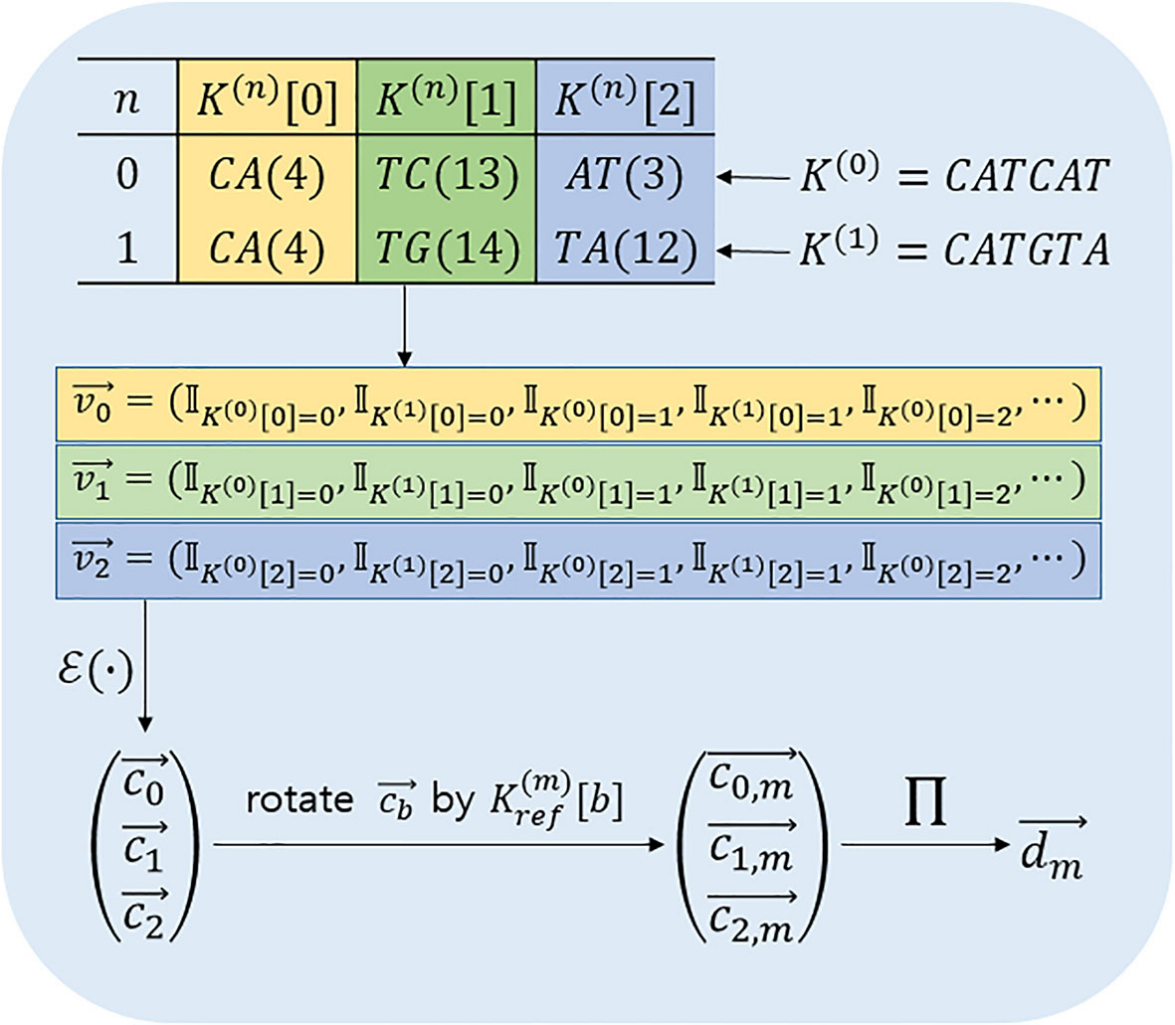
A detailed example to how multiple K-mers are encoded into vectors in parallel. This figure depicts how K-mers are divided into small blocks and then encrypted to vectors. In this example, two 6-mers *K*^(0)^=*CATCAT* and *K*^(1)^=*CATGTA* are encoded into *B*=3 blocks each of size *L*=2 to reduce the size of ciphertext space. Here, the value of slots is a power of 2 bounded by (4^2^)·2. The subscript *b* of $\vec {v}_{b}$ indicates the index of blocks encoded starting from 0. The values of elements in the vectors are indicators to $K^{n}[b]= \frac {i-n}{2}$. In this sense, $\vec {v}_{0}[16]$, $\vec {v}_{0}[17]$, $\vec {v}_{1}[52]$, $\vec {v}_{1}[57]$, $\vec {v}_{2}[12]$, and $\vec {v}_{2}[49]$ are 1’s. The vectors $[\vec {v}_{b}]$ are encrypted into polynomials $[\vec {c}_{b}]$ and then rotated by corresponding value of $K_{ref}^{(m)}$. This rotation ensures that the first *N* blocks indicate the agreement of *Nb*-th block of K-mers and *b*-th block of $K_{ref}^{(m)}$. Later, these values are multiplied in component-wise manner. Therefore, $\vec {d}[n]$ indicates 1 if *n*-th K-mer agrees with $K_{ref}^{(m)}$ in all *B* blocks and 0 if any pair of blocks from both K-mers does not match

HEAAN supports a technique to pack *k* complex numbers in a single polynomial using a variant of the complex canonical embedding map $\phi :\mathbb {C}^{k} \rightarrow \mathcal {R}$. We make use of the technique and encrypt each of $\vec {v}_{b}$’s as a single ciphertext $\vec {c}_{b}$. An example of parallel K-mer encryption is depicted in Fig. 4.

### Homomorphic equality test of K-mers

In the proposed system, encrypted K-mers are compared in homomorphic way to detect SNPs. The evaluation operation consists of following steps.

**Step 1:** The HEAAN scheme supports the rotation operation on plaintext slots, i.e., it enables us to securely obtain an encryption of the shifted plaintext vector (*w*_*r*_,…,*w*_*k*−1_,*w*_0_,…,*w*_*r*−1_) from an encryption of (*w*_0_,…,*w*_*k*−1_). We denote the rotation operation as $\texttt {Rotate}(\vec {c}; r)$. It outputs a ciphertext $\vec {c}_{r}$ encrypting the rotated plaintext vector of $\vec {c}$ by *r* positions. Define and compute

$${\vec{c}}_{b,m} = \texttt{Rotate}\left(\vec{c_{b}}; N\cdot K_{}{ref}^{(m)}[\!b]\right). $$

Note that for *n*∈[ *N*], $\vec {c}_{b,m}[\!n]$ is 1 if *K*^(*n*)^[ *b*]=*Kref*^(*m*)^[ *b*] and 0 otherwise.

**Step 2:** Define and compute

$$\vec{d}_{m} = \prod_{b\in[B]}{\vec{c}_{b,m}}, $$

where $\prod $ denotes component-wise multiplication. Note that for *n*∈[ *N*], $\vec {d}_{m}[\!n]$ is 1 if *K*^(*n*)^=*Kref*^(*m*)^ and 0 otherwise.

**Step 3:** Define and compute

$$\vec{d} = \sum_{m\in[L]}{\vec{d}_{m}}. $$

Note that for *n*∈[ *N*], $\vec {d}[\!n]$ is the number of *m* such that *K*^(*n*)^[ *b*]=*Kref*^(*m*)^[ *b*]. However, it is very unlikely *Kref* to have multiple identical subsequences. We may assume that *Kref* does not have multiple identical subsequences. If this is the case, $\vec {d}[\!n]$ has the value 1 if *K*^(*n*)^=*Kref*^(*m*)^ for some *m* and 0 if not.

**Step 4:** Define and compute

$$\vec{c_{res}} = \sum_{n\in[N]}{ \texttt{Rotate}\left(\vec{d}; n\right)}. $$

Note that the 0th component of $\vec {c_{res}}$ is the encryption of what we wanted: the number of *n* satisfying *K*^(*n*)^=*Kref*^(*m*)^ for some *m*∈[*L*]. The fact can be seen by an easy computation below.

$$\vec{c_{res}}[\!0] = \sum_{n\in[N]}{ \texttt{Rotate}\left(\vec{d}; n\right)[\!0]} = \sum_{n\in[N]}{\vec{d}[\!n]} $$

## Results

### Panel scheme accuracy

As mentioned in the subsection “False positive/negative errors”, the false positive risk of our model can be controlled to some extent by setting a PDT parameter. Therefore, we focused on evaluating the false negative risk by performing an additional experiment. In our model, false negative occurs if a patient has individual sequence variations near the panel SNPs. Unlike the conventional unencrypted mutation-calling procedure allowing few mismatches, our model depends on the equality test process, which always needs perfect matching nearby the panel SNPs. Therefore, even a single unexpected variation neighboring a panel SNP can sabotage the calling of the residue. Considering the prevalent somatic mutations observed in cancer patients, we can consider it as a major source of risk. Therefore, we tested the false negative risk caused by somatic mutations by using residues having mutations in actual breast cancer patients, where the data is provided by TCGA BRCA [40]. By this test, we estimated the empirical probability of false negative risk in various experimental conditions.

We collected 116,607 somatic mutations from TCGA BRCA dataset, then computed the distribution of pairwise distances between two mutations in terms of chromosomal positions. The goal of this test is to estimate the probability that any two mutations are located in proximity by chance. We set the threshold of proximity as 32 bp, which requires at least 32 bp as K-mer size to avoid false positive SNP calling. We will discuss about K-mer size later in detail. For now, the total number of all possible SNP pairs is calculated as follows.

$${{116,607}\choose 2} = {6,798,537,921} $$

and thus the probability *P* of a panel with *N* SNP residues having at least a SNP pair that exists within 32-mer is

1 $$ 1 - \left(1 - \frac{{1,308}}{{6,798,537,921}}\right)^{N\choose 2}.  $$

Table 1 shows the probability of false negative risk calculated by aforementioned formula (Eq. 1). The result indicates that even though the risk gradually increases as the size of panels grows, the false positive risk still remains insignificant even when it reaches to 100 residues. It means that our model can handle panels having large size without significant false negative risk.

**Table 1:** Probability P of two SNPs residing in a 32-mer given N SNP residues

*N*	*P* (%)
10	0.00087
20	0.0037
30	0.0084
40	0.015
50	0.024
60	0.034
70	0.046
80	0.060
90	0.070
100	0.095

N indicates the number of residues selected from 116,607 somatic mutations. P indicates the probability that any two residues are located in 32 bp window. Note that P still remains insignificant even when the N reaches to 100. All numbers are rounded down to 2 significant figures

As shown in Table 1, the false negative risk of our model caused by somatic mutation is not significant. Even though there can be other source of risks that we did not consider such as germline variations, the chance of false negative caused by germline variation is relatively small compared to the one caused by somatic mutation [41*,*^42^]. Although a SNP residue of interest has a sequence variation in its flanking sequence, there are other adjacent K-mers that might still detect the residue. Only when more than two sequence variations are simultaneously occurred and located in K bp on both sides of the panel residue, the scheme completely fails. Hence the actual hazard of false negative is expected to be small in practice. Moreover, even if the false negative error occurs, the system can still know the occurrence of it because there would be no positive count for that SNP residue at all. Therefore, the model can easily detect false negative errors and can recommend to avoid fatal situations.

### Running time of the proposed panel scheme

In our model, one of the major factors that determine running time is the PDT level. As mentioned, PDT level indicates the tolerance for mismatches. Using a higher PDT guarantees less false positive risk, but it increases the length of K-mer i.e., K, which in turn increases the running time. Especially, the homomorphic equality test procedure is significantly affected by K. As shown in Table 2, the running time of homomorphic equality test has almost linear relationship with K (Pearson correlation coefficient of 0.99).

**Table 2:** Length K of product K-mers, time needed to encrypt K-mers and perform equality test to detect SNPs

N	PDT	K	K-mer encryption (ms)	equality test (sec)
10	0	10	45	82
10	1	13	56	141
10	2	17	92	300
10	3	20	101	418
10	4	24	119	603

N indicates the number of SNP residues included in the panel. PDT is the mismatch tolerance, where higher PDT indicates lower false positive risks. The table shows how the changes in PDT and K affect the running time. The result indicates that larger K results in longer running time to encrypt and test equality

With 10 SNP residues and PDT level 4, K is found to be 24 (Table 3). In detail, we have 480 24-mers in the user’s K-mer set and 960 24-mers in the panel K-mer set. The comparison of the two sets took 603 seconds (Table 2). Considering that the PDT level 4 with finding K-mers is overgenerous, despite the wide genome variation our work shows that homomorphic encryption can be computationally feasible to apply on SNP panel scheme.

**Table 3:** The estimated values of K given the number of SNP residues and PDT levels

	*N*=10	20	30	40	50	60	70	80	90	100
PDT = 0	10	21	21	21	21	21	21	21	21	21
1	13	23	23	23	23	23	23	24	24	24
2	17	26	26	26	26	26	26	26	26	26
3	20	27	27	27	27	27	27	28	28	28
4	24	32	32	32	32	32	32	32	32	32

PDT indicates mismatch tolerance and N indicates the panel size. The number inside of table is value of K, which is the minimal length of K-mer for the model. Usually, larger K requires longer running time and more resources. The table shows how the changes in PDT and N affect the value of K. The result indicates that K is positively correlated with both factors respectively

### Panel design guideline for scheme feasibility

The practicality of our scheme relies on shorter K-mers, and minimizing K is one of our best interest. As mentioned in the Method section, K increases as (1) the number of SNP residues included in panels grow and (2) the higher PDT level required. The estimated value of K with the simulated data is shown below (Table 3).

However, the values of K tend to saturate very quickly. There is ignorable difference among the values of K along the number of SNP residues from 20 to 100. The saturating tendency implies that our scheme is likely to be effective even regarding the panel with large number of SNP residues. In the data preprocessing stage, we have discarded SNP residues with redundant flanking sequences to prevent very large number of K. Table 4 shows the number of discarded SNP residues with respect to the initial number of SNP residues of panels. Note that the panels do not drop many SNP residues as their number grows. That is, with carefully chosen SNP residues not to share the redundant flanking sequences, some value roughly around 32 for K would be long enough for any practical number of SNP residues. The value drops to about 21 when PDT is not concerned. Considering the trade-offs these parameters can provide, any user can fully avail oneself to our SNP panel scheme under most circumstances.

**Table 4:** The number of discarded and remaining panels given the original number of SNP residues randomly selected. N indicates the number of residues in each panel

	*N*=10	20	30	40	50	60	70	80	90	100
Total	10	20	30	40	50	60	70	80	90	100
Discarded	0	0	2	3	5	6	7	9	9	13
Remaining	10	20	28	37	45	54	63	71	81	87
Survival rate	1.0	1.0	.93	.93	.90	.90	.90	.89	.90	.87

Discarded indicates the number of residues abandoned during preprocessing. During preprocessing, the model checks if there are residues having too much similarity in their neighboring sequences. If two residues have too much similarity in neighboring sequence contexts, they cannot be distinguished by NGS-seq due to the limited read length. Table shows that remaining rate is consistent even when N reaches 100, which indicates that the model can handle panels with large size without losing too much residues

## Conclusions

Although homomorphic encryption has a good potential for protecting security of data, the encryption method combined with the current computer systems has not achieved practical performance to fully utilize the power. In the field of genomics, the usage of homomorphic encryption has been mostly limited to querying aggregate or annotated data, requiring the preprocessing of data. However, to preprocess the genomic data, the raw genome sequence should be reveled without adequate protection, thus more reliable scheme for protecting the genome information is much needed. In addition, the SNP panel at the hospital should be protected. In this paper, we propose a secure SNP panel scheme that protects both the user’s genome information and the SNP panel information owned by the hospitals. Since the current homomorphic encryption technologies are not computationally efficient, it is not trivial to develop a secure SNP panel scheme that can be used in reality. By chunking the part of genome down into K-mers, we have minimized the size of ciphertext space and overcome the current inefficiency of the homomorphic encryption. The scheme has yet many further possible improvements such as parallel processing and new algorithmic techniques. We expect our method to protect the raw sequence from possible threats and further return the control of genomic data to its owner, and at the same time protect the hospital’s SNP panel assets safely. However, we emphasize that our method shows the feasibility of our scheme. Applying our proposed method to hospitals will certainly require extensive evaluation and improvement.

## Abbreviations

BRCA: Breast cancer susceptibility gene
DNA: Deoxyribonucleic acid
GWAS: Genome-wide association study
HEAAN: Homomorphic encryption for arithmetic of approximate numbers
LWE: Learning with error
NIH: National institutes of health
PDT: Point deviation tolerance
RLWE: Ring-learning with error
SIMD: Single instruction multiple data
SNP: Single-nucleotide polymorphism
TCGA: The cancer genome atlas
UCSC: University of California, Santa Cruz
US: United states
